# Revisiting Eysenck: The Association Between Personality and Acute Stress Reactivity

**DOI:** 10.3390/bs14111098

**Published:** 2024-11-15

**Authors:** Max J. Marshall, Katlyn Peck, Robin Hunter, Julia Totosy de Zepetnek, Alexandra J. Fiocco

**Affiliations:** 1 Institute for Stress and Wellbeing Research, Department of Psychology, Toronto Metropolitan University, 350 Victoria Street, Toronto, ON M5B 2K3, Canada; max.marshall@torontomu.ca (M.J.M.);; 2 Faculty of Kinesiology & Health Studies, University of Regina, Regina, SK S4S 0A2, Canada

**Keywords:** stress reactivity, Trier Social Stress Test, personality, extraversion, neuroticism

## Abstract

Eysenck’s biopsychological model of stress posits that the extraversion–introversion distinction is associated with different thresholds of arousal, which in turn moderate the stress response. Although higher thresholds of activation have been reported in the context of physiological stress induction, more contemporary research has resulted in mixed findings. The objective of the current study was to revisit Eysenck’s biopsychological model of stress by examining associations between the two personality dimensions (extraversion–introversion and neuroticism–stability) and stress reactivity in response to the Trier Social Stress Test. A total of 122 participants aged 18 to 80 years were recruited. Participants completed a battery of questionnaires, including a personality questionnaire, and were exposed to the TSST, during which salivary cortisol and galvanic skin responses (GSR) were recorded. People scoring higher on extraversion displayed heightened GSR relative to people scoring lower on extraversion. Furthermore, sex-based analyses suggest that this association was driven by females. No significant associations were found for cortisol or with respect to the neuroticism–stability dimension of personality. These findings highlight the need to take a more nuanced approach to investigating the association between personality and stress reactivity, highlighting the importance of the stress induction protocol and the stress-sensitive system under investigation.

## 1. Introduction

In 1963, Eysenck proposed that individuals are biologically predisposed toward certain personality traits, and this, in turn, impacts behavior and responses to stress [1]. The stress response involves the activation of two stress-sensitive systems: the hypothalamic–pituitary–adrenal (HPA) axis, and the sympathetic–adrenal–medullary (SAM) axis. The initial, fast-acting SAM axis leads to the release of catecholamines [epinephrine and norepinephrine] and increased respiration, perspiration, and cardiovascular output, which is commonly characterized as the fight-or-flight response [2]. It is through the reticular formation that the hypothalamus signals the brain stem and viscera to activate the sympathetic branch of the autonomic nervous system. The slower-acting, HPA axis results in the culmination of glucocorticoids (i.e., cortisol in humans) from the adrenal cortex, further supporting SAM activity and glucosynthesis to foster energy mobilization [2].

Prior to developing the PEN Model that encompasses three dimensions of personality (a.k.a., super traits) including the obsolete psychoticism–normality dimension, Eysenck proposed that personality mainly consists of two dimensions: extraversion–introversion and neuroticism–stability. The extraversion–introversion dimension reflects the individual’s sociability: extraversion is associated with greater tendencies to be socially engaged and outgoing, whereas introversion is associated with a tendency for shyness and a preference for smaller social groups. Eysenck proposed that the extraversion–introversion distinction is associated with different thresholds of activation of the reticular formation, such that some individuals require greater external stimulation than others to reach an optimal level of physiological functioning [1]. That is, people who score high on extraversion require greater physiological and sensorial stimulation and are more likely to seek more stimulating environments than people who are more introverted. Early studies have examined this hypothesis, including the widely known lemon drop test that showed people scoring high on extraversion displayed lower salivation than introverts when drops of lemon juice were placed on the tongue [3]. This higher threshold of activation in extraverts compared to introverts was also observed in pupillary responses to bright light and brain wave responses to auditory stimuli [4]. The neuroticism–stability dimension refers to the individual’s emotional lability: greater neuroticism is associated with greater emotional lability and negative affect [1]. Eysenck proposed that the neuroticism–stability distinction is associated with differential activation of the limbic system, with greater limbic activation among people who score high on neuroticism [1]. Accordingly, people who score high on neuroticism are more likely to perceive threats and become emotionally aroused, relative to people who score low on neuroticism [5].

While earlier studies examining the association between biological processes (or temperament) and autonomic activity largely focused on physical stimulation (e.g., lemon drop test, visual and auditory stimulation), more recent investigations have examined individual differences in stress physiology across personality traits using psychosocial paradigms, including the Trier Social Stress Test (TSST, [6]). The TSST includes the delivery of a speech and impromptu arithmetic task in front of a panel of study confederates and is one of the most widely used validated psychosocial stressors that taps into the ingredients of stress: novelty, unpredictability, threat to ego, and low sense of control [7]. With a primary focus on evaluative threats, the TSST offers an ecologically valid and relevant tool to examine Eysenck’s biopsychosocial theory of personality. However, there is a paucity of studies that have explored this line of research.

Earlier studies examining associations between Eysenck’s two dimensions of personality and cortisol reactivity to the TSST among undergraduate students reported null associations for both extraversion and neuroticism [8,9]. In a sample of Dutch adolescents who underwent a Leiden Public Speaking Task (a psychosocial stress protocol comparable to the TSST), it was reported that adolescents with higher mean extraversion scores displayed lower cortisol reactivity and that adolescents with higher mean neuroticism scores displayed a stronger SAM response (i.e., pre-ejection period reactivity), both supporting Eysenck’s biopsychological model of stress [10]. Contrary to these findings, Xin et al. [11] found that higher scores on both trait extraversion and neuroticism were associated with a blunted cortisol response in a sample of 54 university students exposed to the TSST. Further, only neuroticism predicted the heart rate response to the TSST, with greater neuroticism associated with lower heart rate reactivity to the TSST. Oswald et al., [12] also reported a blunted cortisol response to the TSST, but in females only. While the limited extant literature highlights the importance of measuring both HPA and SAM axis activation to an acute psychosocial stressor, the discrepancies in study findings require further examination.

A recent meta-analysis of the association between personality and stress was conducted by Luo et al. [13]. The analyses included over 249 studies with a range of stress outcomes, using several measurement tools. Only 20 studies included a physiological outcome, of which 11 examined the association between personality and cortisol and nine studies examined SAM activation, including blood pressure, heart rate, and heart rate variability. Other physiological measures included testosterone, antibodies, and respiratory sinus arrhythmia. The authors reported null to weak associations between personality and physiological outcomes, however, pooling effect sizes across different measurements, sampling protocols, and physiological systems does not provide a reliable or meaningful pooled estimate. Further, the authors did not assess, or address, heterogeneity between studies, which were likely large considering the differences in physiological systems under investigation and their measurement. Although Luo et al. [13] are to be commended for their work, the meta-analysis likely ignored important nuances between the studies (see [14]).

The objective of the current study was to revisit Eysenck’s biopsychological model of stress by examining associations between the two personality dimensions (extraversion–introversion and neuroticism–stability) and stress reactivity, including both SAM and HPA activation, in response to the TSST. Based on Eysenck’s model, it was hypothesized that lower extraversion and greater neuroticism would be associated with greater stress reactivity. Further, given the well-documented sex differences in reactivity to the TSST [15], data were disaggregated to explore associations in males and females separately.

## 2. Materials and Methods

### 2.1. Participants

As part of a larger study, 122 participants aged 18 to 80 years were recruited through online advertisements, flyers, and the university’s participant pools. The objective of the primary study was to examine the effect of acoustic sound on inoculating the stress response [16]. Participants were screened on the telephone and excluded based on the following criteria: self-identifying as a “heavy smoker” (relative to infrequent or light smoker); self-reported use of hormone replacement therapy, antidepressants, anxiolytics, or antipsychotic medications; pregnancy, uncontrolled metabolic disorders, endocrine disorder, seizure disorder, self-reported diagnosis of a psychotic disorder, anxiety or depression. Female participants were asked to report whether they were currently taking an oral contraceptive (yes, no) and their menstrual cycle phase (luteal, follicular, menopause). Irrespective of their response, all female participants were included in the study. Oral contraceptive use and menstrual phase were assessed as potential covariates for models including cortisol as the outcome variable.

This study was approved by the institution’s Research Ethics Board (REB 2015–149). All participants provided written informed consent before participating in this study and were debriefed at the end of the testing protocol.

### 2.2. Measures

#### 2.2.1. Trier Social Stress Test

Stress induction was implemented using the TSST [6], which entailed an anticipation phase and a presentation phase in which the participants gave a 5-min speech on a predetermined topic followed by an unexpected 5-min arithmetic exercise (continuously subtract 13, beginning at 996) in front of a panel of 2 unsympathetic study confederates wearing white lab coats (one male and one female). During the anticipation phase (5 min), participants were told that they were to prepare a 5-min speech to satisfy the “language component” of the study. They were told that two “evaluators” would analyze the content of their speech and that their session would be video recorded for later content analysis. The topic of the speech was based on the age cohort. Younger adults were asked to prepare a 5-min speech on why they are the most qualified person for their future desired job. They were also told that their performance would be compared with age-matched students from another university. Older adults were instructed to prepare a 5-min speech on how they have contributed to society throughout their life and were primed with a stereotype threat that memory and language abilities decline with age. These modifications were made to enhance the socio-evaluate threat of the protocol.

#### 2.2.2. Psychosocial Questionnaires

Participants completed a sociodemographic questionnaire that indexed age, biological sex (0 = female, 1 = male), ethnicity, years of education, and perceived socioeconomic status (0 = very low, 1 = low, 2 = medium, 3 = high, 4 = very high). Participants also completed the Depression, Anxiety, and Stress Scale (DASS-21; [17]), a validated self-report questionnaire designed to assess the symptoms of depression, anxiety, and stress within the last week. To index personality traits, participants completed the NEO-Five Factor Inventory (NEO-FFI; [18]), a 60-item questionnaire developed to measure the five major personality factors of Neuroticism, Extraversion, Openness, Agreeableness, and Conscientiousness. Aligned with Eysenck’s model, analyses included the Extraversion and Neuroticism subscales as predictor variables.

#### 2.2.3. Stress Reactivity Markers

Galvanic skin response (GSR), a marker of sympathetic–adrenal–medullary (SAM) activity, was measured using the BIOPAC BioNomadix wireless system with MP150 data acquisition. GSR, defined as the number of peak responses to the laboratory stressor, was treated as a dependent variable in the analytical models. For full details on this methodology, see Peck et al. [16]. Briefly, two electrodes were placed on the middle and ring finger of the participant’s non-dominant hand and a ground electrode was attached to the middle finger of the non-dominant hand. GSR data was sampled at an acquisition rate of 2000 samples per second, with a gain of 2000. Raw data and regions of interest (i.e., baseline, anticipation, TSST, and four 10-min recovery periods) were visually inspected, filtered for artifacts, extracted, processed, and analyzed using BIOPAC AcqKnowlege 5.0 software.

Cortisol, a marker of HPA activity, was measured in saliva and sampled 8 times over the course of the experimental protocol: baseline (T1, immediately following a 10-min acclimation period and before acoustic listening), following acoustic exposure (T2, immediately following 10-min acoustic listening), following anticipation (T3, following 5-min anticipation and before the speech/arithmetic task), TSST (T4, immediately following the 10-min speech/arithmetic task), and recovery (T5-T8, each at 10-min intervals following T4). Using the passive drool method, samples were collected in 2 mL polypropylene cryovials (Salimetrics, LCC, Carlsbad, CA, USA). Samples were stored in a −70 °C freezer until subsequent in-house analysis using Salimetrics© salivary cortisol enzyme competitive immunoassay standard 96-well plate assay kit. Inter- and intraassay coefficients of variation were 13.8%, and 4.7%, respectively.

### 2.3. Procedure

**Figure 1:**
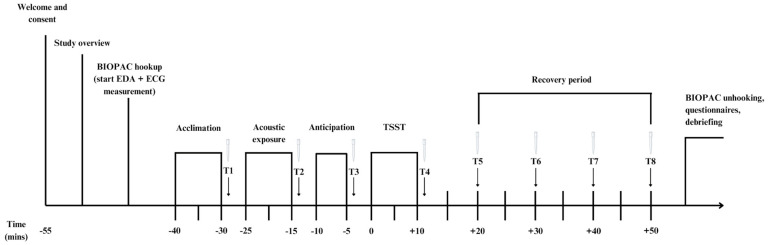
Study Protocol.

Prior to visiting the lab, participants were instructed not to smoke, engage in strenuous physical activity, or consume caffeine or alcohol for a minimum of one hour prior to testing. The study session was 2.5 h long and took place between 1300 h–1800 h. Upon arrival to the lab, participants were fitted with the wireless BIOPAC system and rested for a 10-min acclimatized period. As part of the original study, participants were randomized to one of 3 acoustic conditions; however, acoustic exposure did not impact stress reactivity to the TSST [16]. Following the acclimation period, participants provided a baseline salivary sample (T1), after which they listened to acoustic stimuli for 10 min. Participants then provided a second salivary sample (T2) and underwent the anticipatory phase of the TSST. Participants then provided a third salivary sample (T3) before engaging in the 5-min speech and 5-min arithmetic task. Following task completion, four subsequent salivary samples were collected at 10-min intervals. Following the recovery phase, participants were unhooked from the BIOPAC system and instructed to complete a battery of questionnaires, including the DASS-21 and NEO-FFI, after which they were debriefed (See Figure 1). For full details of the protocol, including acoustic exposure, see [16]. As acoustic exposure did not affect the stress response (with negligible effect size), the acoustic exposure group was not further assessed in the current study. However, to take a more conservative approach, the acoustic group was considered an a priori covariate.

### 2.4. Statistical Analyses

#### 2.4.1. Treatment of Raw Data

Personality scores were standardized, and the data underwent a tertiary split before being entered into the analytical model for ease of interpretation and efficiency. GSR and cortisol data were assessed for assumptions of normality using the Kolmogorov–Smirnov (K-S) test. Outliers were determined using the outlier labeling rule [19] and missing data were assessed using Little’s MCAR (missing completely at random; [20]). Due to the skewness of the data, GRS values were square root transformed, and cortisol values were log-transformed, to approximate a normal distribution. A total of 15 participants were removed from the dataset due to faulty GSR readings that were too noisy to process, which resulted in a final analytical sample of 107 for GSR analyses.

A reactivity score and recovery score were created for both GSR and cortisol. GSR reactivity was defined as the change in GSR from pre-anticipation to post-TSST (T4–T2/T2) and GSR recovery was defined as the change in GSR from the first to final recovery measurement (TSST8–TSST5/TSST5). Since cortisol displays a peak 5 min following the TSST, reactivity and recovery used slightly different measurement points: cortisol reactivity was defined as the change in cortisol from pre-TSST to 5-min post TSST (T5–T3/T3) and cortisol recovery was defined as the change in cortisol from first to final recovery measurement (TSST8–TSST6/TSST6).

#### 2.4.2. Hypothesis Testing

Bivariate correlations were conducted to examine zero-order correlations between all the variables of interest and to assess for potential covariates. To test the study hypotheses, a series of repeated measures analysis of covariance (ANCOVA) were conducted. In each model, the within-subjects variable was either GSR or cortisol, and the between-groups variable was either extraversion or neuroticism. Although the original study failed to show an effect of acoustic sound exposure on stress reactivity, each model controlled for acoustic sound conditions. Additional a priori covariates included age and sex due to the extant literature suggesting their effects on stress reactivity [15,21]. All repeated measures analysis were conducted using Greenhouse–Geisser correction to adjust for violation of sphericity. Significant interactions were further explored with subsequent analyses of the between-group differences at each of the 8 measurement points. Furthermore, ANCOVAs were conducted to determine whether the between-group differences were specific to reactivity or recovery. Finally, exploratory repeated measures ANCOVAs were conducted on sex-based disaggregated data to investigate whether the association between personality and stress reactivity differed in males and females.

## 3. Results

### 3.1. Participant Characteristics

The analytical sample included 107 participants with viable data. The mean age of the sample was 43.14 (SD = 21.83) and 64.5% were female. The participants reported 15.99 (SD = 2.37) years of education on average, and approximately 80% reported medium to very high socioeconomic status. The participant characteristics are presented in Table 1.

### 3.2. Bivariate Correlations

Bivariate correlations revealed that the perceived SES was associated with age and years of education. GSR Stress Recovery was associated with GSR Stress Reactivity. Cortisol Stress Recovery was found to be associated with both years of education and Cortisol Stress Reactivity. Further, Neuroticism was associated with age, ethnicity, and perceived SES. Finally, Extraversion was associated with the perceived SES and Neuroticism. The full correlation matrix is presented in Table 2.

Within females, bivariate correlations suggested that neither menstrual cycle nor oral contraceptive use was associated with stress reactivity or recovery, as measured by GSR or cortisol (*p*-value range 0.213–0.979). Among the 69 female respondents, 21% were in their luteal phase, 25% were in their follicular phase, and 54% were post-menopausal. Among the younger women, 13% were taking an oral contraceptive (7% of the entire female sample).

### 3.3. The Association Between Personality and Galvanic Skin Response to the TSST

**Figure 2:**
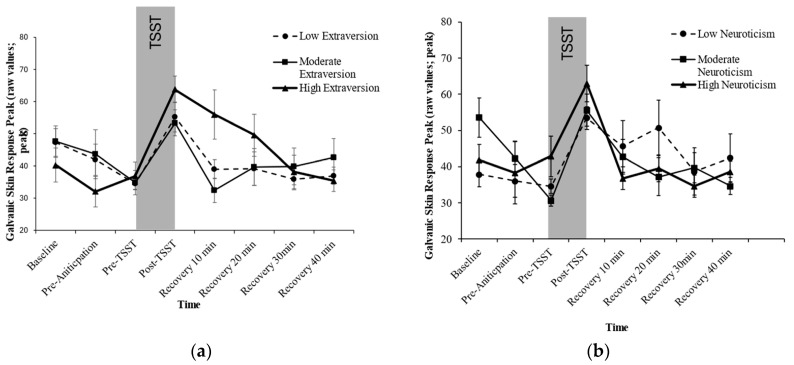
Galvanic Skin Response by Level of Extraversion (**a**) and Neuroticism (**b**).

Controlling for acoustic condition, age, and sex, the analyses revealed a significant Extraversion × Time effect for GSR, *F*(11.418, 439.586) = 2.597, *p* = 0.003, η2 = 0.063 (see Figure 2a). Adjusting for multiple comparisons using Bonferroni adjustment, groups significantly differed at T5 only, with High Extraversion displaying a significantly greater GSR relative to Moderate (Mdiff = 1.825, *p* = 0.001) and Low Extraversion (Mdiff= 1.164, *p* = 0.076).

Controlling for acoustic condition, age, and sex, the ANCOVA revealed a significant effect of Extraversion on GSR stress Reactivity (*F*(2, 79) = 4.19, *p* = 0.019, η2 = 0.096), with High Extraversion expressing the greatest rise in GSR (High = 0.797 (SE = 0.911), Mod = 0.398 (SE = 0.627), Low = 0.322 (SE = 0.556)); and a significant effect of Extraversion on GSR stress Recovery (*F*(2, 80) = 5.591, *p* = 0.005, η2 = 0.123), with High Extraversion expressing slowest recovery to the TSST (High = −0.103(SE = 0.330), Mod = 0.338 (SE = 0.800), Low = −0.015 (SE = 0.215)).

Controlling for acoustic condition, age, and sex, the analyses revealed a significant Neuroticism × Time effect for GSR, *F*(11.006, 429.22) = 2.164, *p* = 0.015, η2 = 0.053 (see Figure 2b). Adjusting for multiple comparisons using Bonferroni adjustment, groups significantly differed at baseline (T1) only, with Moderate Neuroticism displaying a significantly greater GSR relative to Low Neuroticism (Mdiff = −1.480, *p* = 0.01) but did not differ from High Neuroticism (Mdiff= −0.583, *p* = 0.867).

Controlling for acoustic condition, age, and sex, the ANCOVA failed to show statistically significant associations with respect to Neuroticism and GSR reactivity (*F*(2, 80) = 1.576, *p* = 0.213, η2 = 0.038) and GSR recovery (*F*(2, 81) = 1.570, *p* = 0.855, η2 = 0.004).

### 3.4. The Association Between Personality and Cortisol Reactivity to the TSST

**Figure 3:**
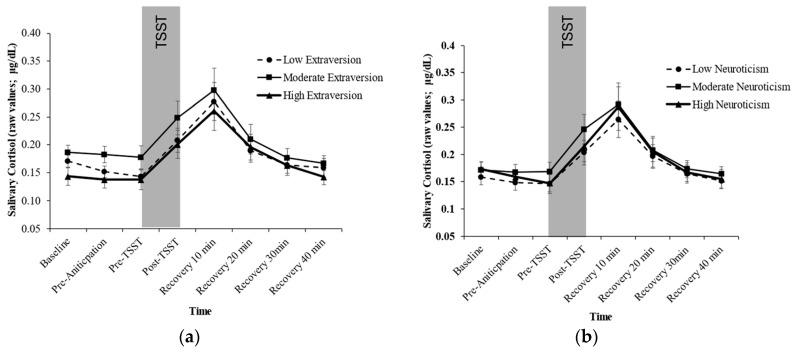
Cortisol Response by Level of Extraversion (**a**) and Neuroticism (**b**).

Controlling for acoustic condition, age, and sex, the analyses failed to show a significant Extraversion × Time effect for cortisol, *F*(4.804.418, 220.985) = 0.988, *p* = 0.464, η2 = 0.021). Similarly, controlling for acoustic condition, age, and sex, the analyses failed to show a significant Neuroticism × Time effect for cortisol, *F*(4.709, 218.991) = 0.224, *p* = 0.945, η2 = 0.005) (See Figure 3). Including oral contraceptives or menstrual cycle as covariates did not change the results.

Controlling for acoustic condition, age, and sex, the ANCOVA failed to show a significant association between Extraversion and Cortisol Reactivity (*F*(2, 94) = 0.170, *p* = 0.844, η2 = 0.004) or Cortisol Recovery (*F*(2, 92) = 1.183, *p* = 0.311, η2 = 0.025). Similarly, no statistically significant associations were found between Neuroticism and Cortisol Reactivity (*F*(2, 95) = 0.212, *p* = 0.809, η2 = 0.004) or Cortisol Recovery (*F*(2, 93) = 0.314, *p* = 0.732, η2 = 0.007).

### 3.5. Exploratory Disaggregation by Sex

In females, controlling for acoustic condition and age, the repeated measures analysis revealed a significant Extraversion × Time effect (*F*(11.203, 257.676) = 2.349, *p*= 0.009, η2 = 0.093) and a significant Neuroticism × Time effect (11.188, 262.909) = 2.537, *p*= 0.004, η2 = 0.097) for GSR. No significant interactions were found for change in cortisol over time with respect to Extraversion (*F*(4.549, 129.639) = 1.006, *p* = 0.413, η2 = 0.034) or Neuroticism (*F*(4.443, 128.514) = 0.229, *p* = 0.935, η2 = 0.008) in females.

In females, ANCOVAs showed a significant association between Extraversion and GSR Reactivity (*F*(2, 47) = 4.498, *p* = 0.016, η2 = 0.161). Females in the High Extraverion tertile displayed the largest rise in GSR relative to the moderate and low tertile groups (High = 0.681 (SE = 0.652), Mod = 0.332 (SE = 0.127), Low = 0.093 (SE = 0.083); however, no significant association was found for the association between Extraversion and GSR Recovery (*F*(2, 49) = 2.113, *p* = 0.132, η2 = 0.079), Neuroticism and GSR Reactivity (*F*(2, 48) = 0.311, *p* = 0.734, η2 = 0.013), or Neuroticism and GSR Recovery (*F*(2, 50) = 1.178, *p* = 0.316, η2 = 0.045). Finally, all ANCOVAs examining the relationship between personality and Cortisol Reactivity and Cortisol Recovery were not statistically significant in females (*p*-level range = 0.381–0.854).

In males, controlling for age and acoustic condition, the repeated measures analysis failed to show a significant Extraversion × Time effect (*F*(9.710, 131.091) = 1.198, *p*= 0.299, η2 = 0.081) or a significant Neuroticism × Time effect (*F*(9.382, 126.654) = 1.353, *p* = 0.214, η2 = 0.091) for GSR. Similarly, no significant Extraversion × Time effect (*F*(4.904, 76.090) = 1.467, *p* = 0.211, η2 = 0.086) or Neuroticism × Time effect (*F*(4.843, 75.073) = 0.281, *p* = 0.918, η2 = 0.018) was found for cortisol.

In males, ANCOVAs failed to show a significant association between Extraversion and GSR Reactivity (*F*(2, 28) = 0.907, *p* = 0.415, η2 = 0.061); however, Extraversion was significantly associated with GSR recovery (*F*(2, 27) = 4.827, *p* = 0.016, η2 = 0.263), such that males in the high Extraversion tertile displayed the steepest GSR recovery and males in the moderate tertile displayed a rise in GSR during the recovery period (High = −0.237 (SE = 0.080), Mod = 0.414 (SE = 0.210), Low = 0.001 (SE = 0.057)). In contrast, a marginal association was found between Neuroticism and GSR Reactivity (*F*(2, 28) = 3.223, *p* = 0.055, η2 = 0.187), and a null association was found between Neuroticism and GSR Recovery (*F*(2, 27) = 1.514, *p* = 0.238, η2 = 0.101. Finally, all ANCOVAs examining the relationship between personality and Cortisol Reactivity and Cortisol Recovery were not statistically significant in males (*p*-level range: 0.347–0.861)

## 4. Discussion

Personality and temperament are integral components of an individual’s psychological makeup, influencing how they perceive and interact with the world. Personality encompasses enduring patterns of thoughts, emotions, and behaviors that characterize an individual, reflecting their uniqueness and consistency across various situations. The objective of the current study was to revisit Eysenck’s biopsychosocial model of stress and examine whether the model’s two dimensions of personality (extraversion–introversion and neuroticism–stability) were associated with stress reactivity. In line with Eysenck’s model, it was hypothesized that both greater neuroticism and lower extraversion would predict greater reactivity to stress.

The current study findings failed to support Eysenck’s conclusions that extraversion is associated with lower physiological reactivity relative to introversion. Rather, people who scored higher in extraversion displayed a greater sympathetic stress response to the TSST and also displayed a notably elevated GSR during recovery. These findings are aligned with contemporary research that suggests a more dimensional model of the association between extraversion and stress reactivity, particularly with respect to the potency of the stressor. Lü et al. [22] proposed that the relationship between extraversion and stress reactivity is affected by the potency of the stressor, reporting that higher extraversion promoted lower cardiovascular reactivity after moderate intensity social stress, but higher stress reactivity when the intensity of the social stress was high. More specifically, they suggested that since social proficiency is higher among people who are highly extraverted, they may be more actively responsive to the stressor. That is, when the demands of the social stressor become greater, highly extraverted individuals become more reactive and more aware of the stressor given their higher sociability. The TSST is designed to effectively elicit the common ingredients that define stressors, namely novelty, low sense of control, unpredictability, and socio-evaluative threat [7]. In the current study, the ego-threat component was modified by including a social comparison element (i.e., noting performance comparisons with other groups). Consequently, the potency of the social threat may have been strong enough that it invoked increased reactions from those higher in extraversion, aligning with the results by Lü et al. [22]. These findings offer evidence for a more dimensional relationship between stress reactivity and extraversion, which imposes nuances in Eysenck’s model.

Study hypotheses with respect to neuroticism were not supported. Although the overall statistical model suggested a group-by-time interaction, groups only differed at baseline, with people scoring moderately on neuroticism displaying the highest electrodermal activity. Although the high neuroticism group displayed higher electrodermal activity during the TSST, the difference was not statistically significant. The extant literature examining the association between neuroticism and stress reactivity has been largely mixed, with some studies reporting a blunted stress response (e.g., [23,24,25]) and others reporting null associations (e.g., [26]). A major limitation in the field is the lack of harmonization across studies with respect to sample characteristics (e.g., age, sex) and stress induction paradigms (e.g., TSST, mental challenge; virtual exposure vs. in-person), making inter-study comparisons and interpretation challenging.

Null associations regarding the association between personality and salivary cortisol reactivity to the TSST are consistent with previous studies employing the TSST as the stress induction protocol [8,9,27]. However, studies using alternative stress induction protocols have reported associations between stress reactivity and personality traits, neuroticism, and extraversion. Employing the Paced Auditory Serial Addition Test (PASAT), a mental arithmetic stressor, people scoring higher on neuroticism secreted lower salivary cortisol in response to the stressor, suggesting a blunted response [25]. Furthermore, in a sample of young adults, people scoring low on neuroticism displayed a significantly greater plasma cortisol response following a combined dexamethasone–corticotropin-releasing hormone test, relative to people scoring high on neuroticism [28]. These findings may suggest that people with high neuroticism may present with a downregulated HPA axis as a protective mechanism, which may serve adaptation and survival. With respect to the introversion-extraversion continuum, greater extraversion has been found to correlate with lower salivary cortisol reactivity in adolescents [10].

Bivariate correlations failed to show statistically significant associations between GSR and cortisol reactivity, or recovery. Null correlations may create speculation that one or both measures are invalid estimates of stress reactivity. However, null bivariate correlations may also be explained by the impact of sampling time on biological estimates of stress. For example, the time it takes to sample salivary cortisol does not exactly overlay the time stamps for each GSR measurement. Furthermore, given the time delay in cortisol reactivity, relative to sympathetic activity, calculations for reactivity and recovery differed for estimates of GSR and cortisol reactivity and recovery.

The current study also explored sex-specific associations. Although medium effect sizes were found for females, the small number of males in the sample most likely diluted the effects of TSST in males. However, when narrowing the window of measurement to the period of recovery, males in the highest Extraversion tertile displayed the steepest recovery. Given this small sample size, suggestions cannot accurately be drawn from these data. Future research will benefit from examining potential sex differences in males and females with more equal distribution across samples. Considering the robust moderating effect of sex on stress reactivity [15,29,30,31], it is important to examine the relative contribution of personality in males and females separately.

Future research is needed to better understand the nuanced association between personality and physiological stress reactivity. While a relatively robust association has been drawn from studies examining perceptions of stress, associations with physiological markers of stress remain unclear [13]. This is largely due to faulty assumptions that result from comparing apples and oranges, so to speak. HPA and SAM are distinct stress-sensitive systems that cannot be merged as a single marker of stress physiology. Furthermore, laboratory protocols used to induce stress largely differ across studies and require greater standardization across labs. Research suggests that HPA and SAM may respond differently, depending on the stress protocol [32]. Accordingly, additional studies are needed to determine the association between personality and stress reactivity across multiple systems and stress protocols.

While the present study contributes to the extant literature, study limitations should be considered. First, this study is a secondary analysis of data that was collected for a larger study that examined music listening on stress inoculation before stress exposure. Although the study failed to show an effect of music treatment on stress physiology, it may be argued that differential acoustic exposures before the TSST may have had confounding effects. However, all analyses in the current study controlled for the acoustic group and likely did not influence the results as the acoustic group did not predict variance in the outcome measures. A second limitation to consider is the potency of the stressor. Differential stress reactivity by personality traits may be washed out if the stress induction protocol is particularly stressful, this is especially true if personality traits account for a small portion of the variance in stress reactivity and recovery. Future research is needed to investigate the relative association between personality and stress induction paradigm. Although the choice of stress induction protocol is often chosen based on resources (e.g., funding, highly qualified personnel), it is unreasonable to assume that all stress protocols are created equal, eliciting the same expected stress response across sympathetic and neuroendocrine systems. Exploring the relative associations across several stress induction protocols will also help to elucidate the association between personality and stress reactivity given mixed findings in the literature. Finally, it may be argued that the current sample was not powered to robustly detect small effects. Furthermore, the relatively smaller sample of males relative to females did not allow for robust moderation analyses by sex. As such, future research should consider powering studies based on very small effect sizes and stratifying their sampling method by sex.

## 5. Conclusions

This study contributes to the literature by revisiting Eysenck’s model of personality and stress reactivity in a contemporary context. The findings highlight the need to take a more nuanced approach to investigating the association between personality and stress reactivity, highlighting the importance of the stress induction protocol and the stress-sensitive system under investigation.

## Figures and Tables

**Table 1:** Sample Characteristics (N=107).

Descriptive	Mean (SD, Range) or % (n)
Age (years)	43.15 (21.83, 18–80)
Education (years)	15.99 (2.37)
Sex (%female)	64.50 (69)
Ethnicity	
European/White	51.40 (55)
African American/Black	5.61 (6)
Hispanic/Latin American	7.48 (8)
Middle Eastern	1.87 (2)
Asian/Southeast Asian	15.89 (17)
South Asian	12.15 (13)
Other/Mix	4.67 (5)
Perceived SES	
Very low	2.8 (3)
Low	18.69 (20)
Medium	64.49 (69)
High	11.21 (12)
Very high	1.87 (2)
Extraversion Score	50.00 (11.67, 25.00–75.00)
Neuroticism Score	49.00 (12.41, 25.00–74.00)

**Table 2:** Correlation Matrix (Kendall’s Tau b).

Variable	1	2	3	4	5	6	7	8	9	10
1. Age	--									
2. Sex (0 = female; 1 = male)	0.210 *	--								
3. Ethnicity	0.333 **	−0.159	--							
4. Education (years)	0.307 **	0.117	−0.098	--						
5. Perceived SES	0.311 ***	0.042	−0.065	0.216 **	--					
6. GSR Stress Reactivity	0.168 *	0.080	−0.113	0.041	0.031	--				
7. GSR Stress Recovery	0.098	0.150	−0.008	0.127	0.099	0.176 *	--			
8. Cortisol Stress Reactivity	0.057	−0.040	0.022	0.118	0.034	0.052	−0.009	--		
9. Cortisol Stress Recovery	0.090	0.009	−0.032	0.170 *	0.058	0.059	0.027	0.629 **	--	
10. Neuroticism	0.323 ***	0.002	0.218 **	−0.035	−0.268 ***	0.022	0.034	−0.004	0.052	--
11. Extraversion	−0.003	−0.197 *	0.073	0.043	0.175 *	0.081	0.044	0.008	0.004	−0.173 *

* Correlation is significant at the 0.05 level (2-tailed). ** Correlation is significant at the 0.01 level (2-tailed). *** Correlation is significant at the 0.001 level (2-tailed).

## Data Availability

Data pertaining to this study may be found on the Open Science Framework (pending).
